# Assessment of ability of a DNA language model to predict pathogenicity of rare coding variants

**DOI:** 10.1038/s10038-025-01385-3

**Published:** 2025-08-15

**Authors:** David Curtis

**Affiliations:** UCL Genetics Institute, University College London, London, UK

**Keywords:** Predictive markers, Medical genetics

## Abstract

A recently described method to predict pathogenicity of DNA variants uses a DNA language model and can be applied to both coding and non-coding variants. For coding variants the performance of this method, termed GPN-MSA (genomic pretrained network with multiple-sequence alignment), was reported to be superior to CADD. We compare the performance of this method against 45 other predictors applied to rare coding variants in 18 gene-phenotype pairs. We find that while GPN-MSA produces stronger evidence for association than CADD it is not the best-performing method for any gene and on average other prediction methods are superior. While GPN-MSA may be useful for predicting the pathogenicity of non-coding variants, it would seem sensible for clinicians and researchers to utilise other methods when dealing with coding variants.

This research has been conducted using the UK Biobank Resource.

## Introduction

A key issue in genetics research and clinical practice is to predict the deleteriousness of nonsynonymous variants. We recently reported a systematic comparison of 45 such predictors, using weighted burden rare variant analysis of exome sequence data applied to 18 gene-phenotype pairs [1].

A new study describes a deleteriousness prediction score based on a DNA language model termed GPN-MSA (genomic pretrained network with multiple-sequence alignment) [2]. Since this is based only on DNA sequence it can be applied to both coding and non-coding variants and the authors reported that it performs well compared to the CADD predictor when applied to nonsynonymous variants [3].

Here, we extend our previous assessment of predictors of pathogenicity of nonsynonymous variants in order to include GPN-MSA alongside the other predictors.

## Materials and methods

The methods used are essentially the same as those described in the previous study [1].

### Dataset

The UK Biobank Research Analysis Platform was used to access the Final Release Population level variants for 469,818 exomes obtained using the protocols described here: https://dnanexus.gitbook.io/uk-biobank-rap/science-corner/whole-exome-sequencing-oqfe-protocol/protocol-for-processing-ukb-whole-exome-sequencing-data-sets [4]. UK Biobank had obtained ethics approval from the North West Multi-centre Research Ethics Committee which covers the UK (approval number: 11/NW/0382) and had obtained written informed consent from all participants. The UK Biobank approved an application for use of the data (ID 51119) and ethics approval for the analyses was obtained from the UCL Research Ethics Committee (11527/001).

### Variant annotation

Attention was restricted to rare variants with minor allele frequency (MAF) <= 0.01. Variants were annotated using Variant Effect Predictor (VEP) [5]. Variants annotated as stop gained, frameshift and essential splice site were given a score of 1 for the loss of function (LOF) category while variants annotated as protein altering, missense, start lost or stop lost were given a score of 1 for the protein altering category. Variants not included in either the LOF or protein altering category were not considered further in these analyses.

The scores for GPN-MSA for all possible single DNA base changes were downloaded from the website provided by the study authors https://huggingface.co/datasets/songlab/gpn-msa-hg38-scores. The score provided consists of the logarithm of the likelihood ratio for the ALT allele compared to the REF allele, so before use the score was multiplied by -1 so that a high score would indicate an ALT allele with a lower likelihood and hence with higher predicted pathogenicity. In order to obtain scores using AlphaMissense, VEP was run with the options *b --canonical –regulatory --plugin AlphaMissense* [6]. This produces two AlphaMissense annotations, a raw score and a categorisation of likely pathogenic, likely benign or ambiguous. These three categories were converted to numerical scores of 2, 0 or 1 respectively and this was used, termed the prediction score, as well as the raw score. To obtain scores for other predictors, dbNSFP v4 was used [7]. For the nonsynonymous and splice site variants listed in dbNSFP v4, scores were obtained consisting of the rank scores for a variety of different prediction and conservation methods. A total of 43 such scores were used, as presented below and as detailed at http://database.liulab.science/dbNSFP.

For each variant and each of these prediction methods, the pathogenicity score was multiplied by a weight based on allele frequency, with rarer variants having higher weights [8]. The LOF or protein altering score for each variant was also multiplied by the weight based on allele frequency. For each gene, an individual would be assigned an overall score consisting of the sum of the relevant scores for the variants carried by that individual, meaning that each individual received an overall LOF score and protein altering score as well as 46 scores for the 46 different pathogenicity predictors evaluated. The GENEVARASSOC and SCOREASSOC programs were used to obtain these scores [9].

### Gene-phenotype pairs

The gene-phenotype pairs selected for this study are shown in Table 1 and consisted of those which had previously produced exome-wide significant results in weighted burden analyses using phenotypes of hypertension, hyperlipidaemia and type 2 diabetes [10–12]. For each phenotype, a mixture of self-report, recorded diagnoses and medication reports was used to designate a set of participants as cases, with all other participants taken to be controls. There were a total of 469,818 exome-sequenced UK Biobank participants, of whom 167,127 were designated cases for hypertension, 106,091 for hyperlipidaemia and 33,629 for type 2 diabetes. As noted in the previous report, for some genes variants predicted to impair function were protective and were associated with lower risk of developing the clinical phenotype [1]. Thus, rare damaging variants in these genes were positively associated with being a control rather than being a case. For the purpose of the current study, in order to make it easier to interpret the results for these genes alongside the others, the phenotype of interest for these genes is taken to be “being a control”.

**Table 1:** SLPs produced by GPN MSA and the ten other prediction methods which yielded the highest average SLP across all genes

Phenotype	Hyperlipidaemia						Hypertension								Type 2 diabetes				
Prediction method	*LDLR* (case)	*ABCG5* (case)	*NPC1L1* (control)	*PCSK9* (control)	*APOC3* (control)	*ANGPTL3* (control)	*DNMT3A* (case)	*FES* (case)	*ASXL1* (case)	*SMAD6* (case)	*NPR1* (case)	*GUCY1A1* (case)	*INPPL1* (control)	*DBH* (control)	*GCK* (case)	*HNF4A* (case)	*HNF1A* (case)	*GIGYF1* (case)	Average
GPN-MSA	***16.91***	1.97	0.46	2.36	0.90	1.23	–0.03	0.98	–0.94	–0.79	0.28	1.21	0.41	**4.11**	**4.38**	1.41	1.59	0.51	2.05
AlphaMissense Score	***80.06***	1.56	0.73	***11.61***	–0.15	2.59	2.97	**4.34**	0.32	0.44	**4.15**	**4.40**	1.41	1.84	**4.36**	***8.24***	**3.72**	2.11	***7.48***
AlphaMissense Category	***76.32***	1.99	0.71	***9.66***	–0.45	**3.91**	2.93	2.98	0.44	0.24	**4.81**	**4.44**	1.16	2.58	***6.49***	***6.15***	2.27	1.16	***7.10***
Polyphen2 HVAR rankscore	***50.16***	1.41	0.79	***7.09***	0.51	0.40	0.42	***6.85***	0.11	0.70	2.41	2.71	1.30	**3.91**	***10.21***	2.19	1.41	0.33	**5.16**
Polyphen2 HDIV rankscore	***44.08***	1.03	1.35	***6.23***	1.05	0.26	1.04	***6.90***	0.17	0.41	0.96	1.90	0.83	**3.06**	***10.61***	2.36	1.28	0.22	**4.65**
SIFT4G converted rankscore	***36.24***	0.34	1.49	**4.84**	–0.02	0.58	**3.45**	**3.53**	0.46	0.46	**3.96**	**3.44**	−0.05	**5.59**	***11.14***	**3.08**	0.44	0.29	**4.40**
MutationAssessor rankscore	***45.84***	1.28	1.43	***6.34***	0.00	1.88	1.51	1.96	−0.15	0.42	**3.66**	0.00	0.69	1.67	***8.19***	2.67	0.00	0.00	**4.30**
SIFT converted rankscore	***27.01***	1.22	1.05	***10.07***	0.02	1.30	0.53	**4.07**	−0.06	0.45	**3.66**	**4.20**	0.21	**5.15**	***10.08***	2.74	0.13	0.35	**4.01**
PROVEAN converted rankscore	***31.41***	1.06	1.39	***9.75***	0.40	2.03	0.48	1.72	0.33	0.11	2.49	**3.33**	-0.18	**3.90**	***6.24***	**4.11**	0.73	0.04	**3.85**
VEST4 rankscore	***33.49***	1.42	0.73	**4.05**	–0.35	0.67	1.14	**3.43**	0.09	0.22	0.74	2.07	0.41	2.48	***9.21***	**3.14**	1.37	0.19	**3.58**
LRT converted rankscore	***35.81***	1.68	0.46	**5.80**	0.23	–0.63	1.45	**3.13**	−0.05	−0.52	0.23	1.71	0.21	2.37	***6.84***	**3.99**	1.06	0.45	**3.57**

SLPs of 3 or more are shown in bold and SLPs of 6 or more in bold italics. The final column shows the mean SLP achieved by each predictor across all genes. Below each gene is an indication of whether damaging rare variants are associated with case or control status for the relevant phenotype

### Comparison of pathogenicity predictors

To gain an understanding of the relationships between the different prediction methods, a correlation matrix was produced of their scores across all the variants annotated as missense by VEP in all the genes and this matrix was visualised using the correl package in R [13, 14].

In order to assess the relative performance of the pathogenicity predictors, for each gene logistic regression analysis was carried out with the relevant phenotype as the outcome and using a model including 20 population principal components and sex as covariates along with the LOF score, protein altering score and predictor score. The Wald statistic was used to obtain the p value for the predictor score and this was converted into a signed log p value (SLP), consisting of the logarithm base 10 of the p value and given a positive sign if the score was positively correlated with the phenotype in question. For each gene-phenotype pair, this process was repeated 46 times to obtain an SLP for each prediction method.

Data manipulation and statistical analyses were performed using GENEVARASSOC, SCOREASSOC and R [9, 14, 15].

## Results

### Correlations between pathogenicity predictor scores

**Fig. 1:**
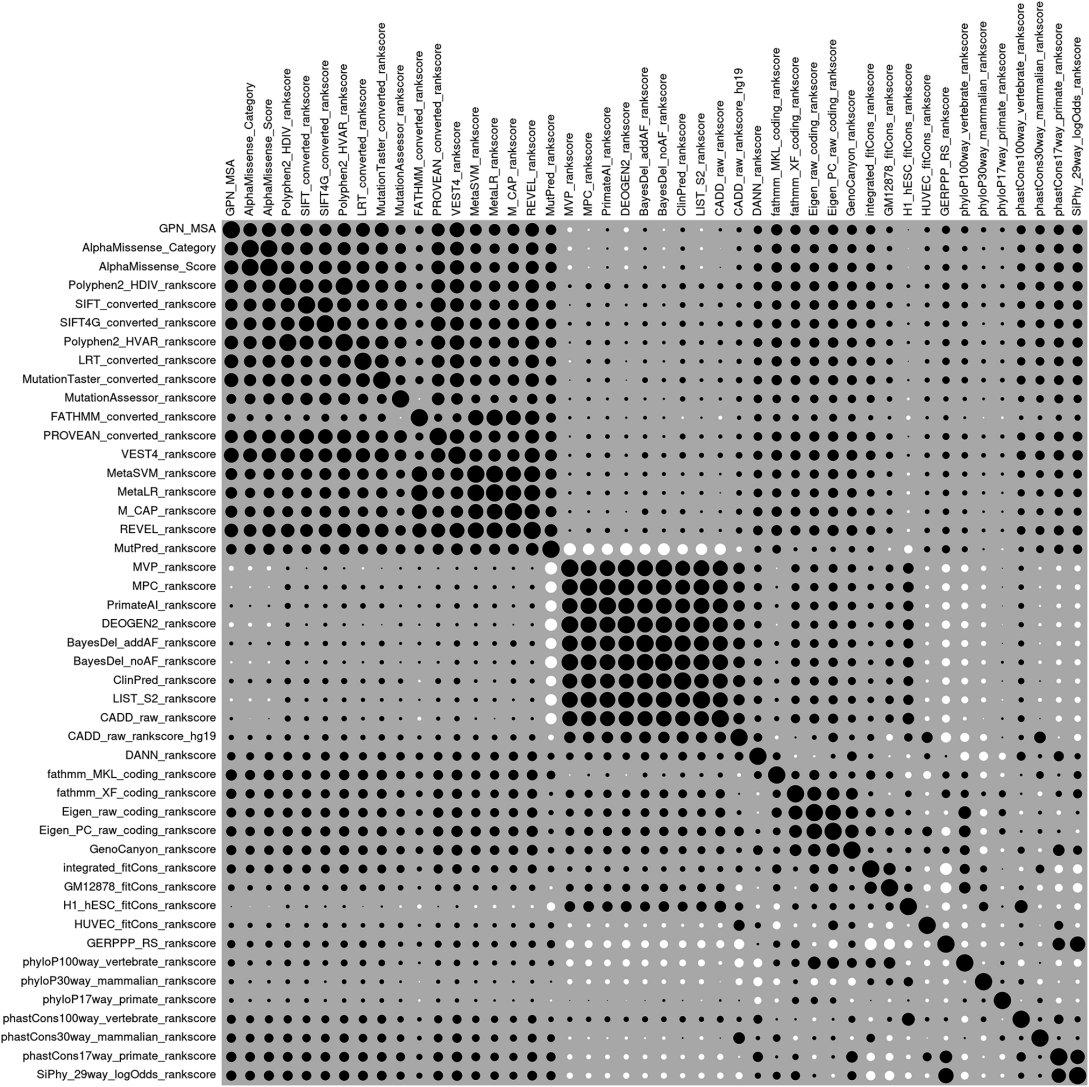
Heatmap showing pairwise correlations between predictor scores across 9568 variants annotated as missense. Black circles indicate positive correlations and white circles negative correlations

In order to gain insights into the relationships between the predictors, pairwise correlation coefficients were obtained between all pairs across variants annotated as missense in all genes, comprising 9568 variants, and a heatmap illustrating these correlations is shown in Fig. 1. The raw correlation coefficients themselves are tabulated in Supplementary Table 1. It can be seen that GPN-MSA, in the first row and column, is positively correlated with a number of other predictors and falls in a block which includes AlphaMissense, SIFT and PolyPhen [6, 16, 17]. As noted in the previous analysis, other predictors have scores which show little or no correlation with the scores produced by these predictors, indicating that the different prediction algorithms can produce markedly different results.

### Performance comparison of pathogenicity predictors

**Fig. 2:**
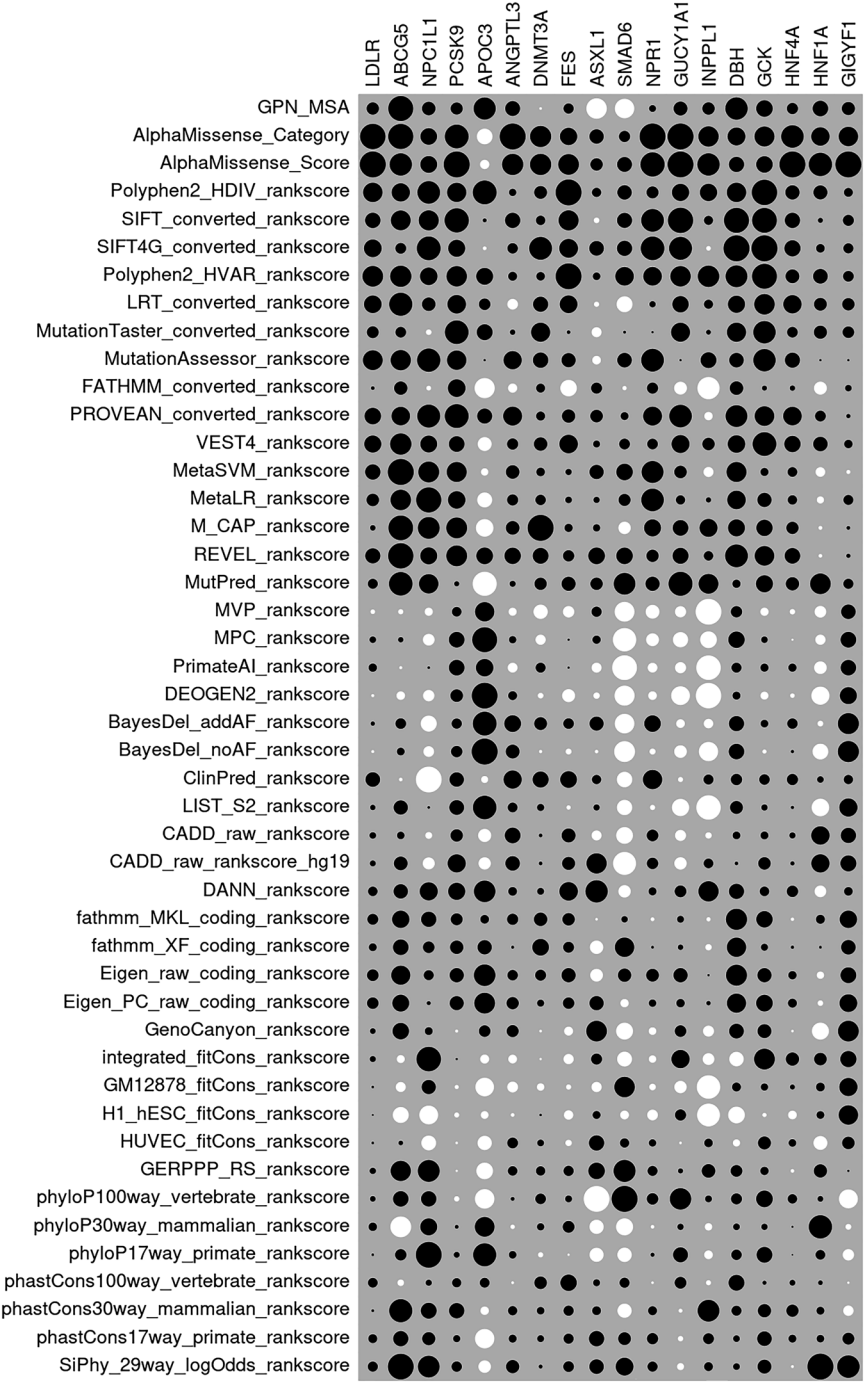
Heatmap of SLPs produced by each prediction method for each gene. The sizes of the dots for each gene are proportional to the SLP for each annotation relative to the maximum SLP produced by any annotation for that gene. White circles indicate negative SLPs

Figure 2 shows a heatmap which illustrates the relative magnitude of the SLP produced by each predictor for each gene and the SLPs themselves are presented in Supplementary Table 2. Table 1 shows the SLPs for GPN-MSA and for the ten other predictors which have the highest average SLP across all genes. As reported in the previous analysis, there is considerable variability in performance of different methods and no method consistently generates high SLPs across all genes. There is no gene for which GPN-MSA yields the highest SLP and across all genes it produces an average SLP of 2.05 compared to the AlphaMissense score which produces an average of 7.48. However GPN-MSA does produce a similar SLP to AlphaMissense for *GCK*, though PolyPhen produces a much higher SLP. Interestingly, for *DBH* GPN-MSA produces an SLP of 4.11 while the AlphaMissense score only yields an SLP of 1.84 (although SIFT4G produces and SLP of 5.59). CADD, to which GPN-MSA was previously compared, was not among the top ten predictors and in fact yielded an average SLP of only 0.76.

## Discussion

When applied to this dataset, GPN-MSA does demonstrate an ability to identify variants which are more likely to be pathogenic in some of the genes studied and its performance overall is better than that of CADD. However, in general its performance is inferior to a number of other methods which have been specifically developed to predict the pathogenicity of nonsynonymous variants. We note that the phenotypes studied here are common and we do not know what the relative performance of these methods would be for identifying variants causing rare Mendelian diseases.

From a theoretical machine-learning point of view, it is of some interest that a method trained to recognise pathogenicity of variants across the genome has inferior performance for nonsynonymous variants than methods which have been specifically developed to assess the effects of amino acid changes. Presumably GPN-MSA may to some extent “recognise” when it is dealing with coding variants but if so this is not sufficient to overcome the effects of also being trained on non-coding variants.

In practical terms, researchers and clinicians analysing both coding and non-coding variants might consider using GPN-MSA to predict pathogenicity of non-coding variants while applying other methods when dealing with coding variants.

## Supplementary information

Supplementary Tables

## Data Availability

The raw data is available on application to UK Biobank.

## Code availability

Scripts and relevant derived variables will be deposited with UK Biobank. Software and scripts used to carry out these analyses are also available at https://github.com/davenomiddlenamecurtis.
